# Broad, broader, broadest

**DOI:** 10.1007/s12471-015-0679-4

**Published:** 2015-04-11

**Authors:** L.X. van Nunen, S.A.W.G. Dello, L.R.C. Dekker

**Affiliations:** Department of Cardiology, Catharina Hospital Eindhoven, Michelangelolaan 2, 5623 EJ Eindhoven, The Netherlands

## Question

**Fig. 1:**
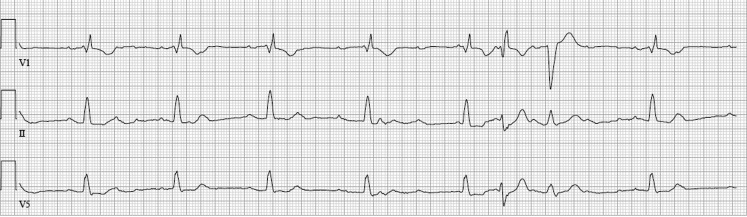
ECG at presentation showing third-degree AV block

A 79-year-old woman was admitted to the emergency department because of respiratory tract infection and several near-syncopes due to intermittent third-degree AV block (Fig. 1). She had a medical history of known paroxysmal atrial fibrillation without left ventricular dysfunction, and chronic mental depression treated by oral flecainide and nortriptyline, respectively.

**Fig. 2:**
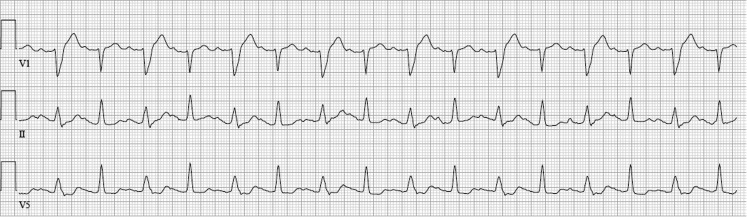
ECG 2 h after presentation

**Fig. 3:**
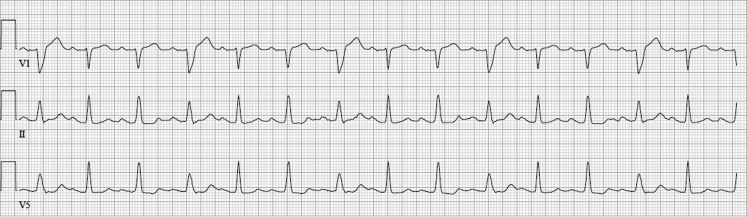
ECG 3 h after presentation

An underlying respiratory tract infection (C-reactive protein 150 mg/l) with a right lower lobe infiltrate was discovered on X-ray. A temporary pacemaker lead was placed and the patient was admitted to the cardiac care unit. Flecainide and nortriptyline were stopped at admission. Over the next hours, the AV block disappeared and the following subsequent electrocardiograms were recorded (Figs. 2 and 3).

What is your diagnosis and can you explain the underlying mechanism?

